# Thermal neutron transmutation doping of GaN semiconductors

**DOI:** 10.1038/s41598-020-72862-2

**Published:** 2020-10-01

**Authors:** R. Barber, Q. Nguyen, J. Brockman, J. Gahl, J. Kwon

**Affiliations:** 1 Department of Electrical Engineering and Computer Science, University of Missouri, Columbia, MO 65201 USA; 2 University of Missouri Research Reactor, University of Missouri, Columbia, MO 65201 USA

**Keywords:** Electronic properties and materials, Experimental nuclear physics, Semiconductors, Electronic devices

## Abstract

High quality Ge doping of GaN is demonstrated using primarily thermal neutrons for the first time. In this study, GaN was doped with Ge to concentrations from 10^16^ Ge atoms/cm^3^ to 10^18^ Ge atoms/cm^3^. The doping concentrations were measured using gamma-ray spectroscopy and confirmed using SIMS analysis. The data from SIMS analysis also show consistent Ge doping concentration throughout the depth of the GaN wafers. After irradiation, the GaN was annealed in a nitrogen environment at 950 °C for 30 min. The neutron doping process turns out to produce spatially uniform doping throughout the whole volume of the GaN substrate.

## Introduction

In 2005, approximately 30% of electrical energy in the United States passed through power electronic converters. It is estimated that this number will increase to 80% by 2030^1^. As these needs increase, the demand for more efficient power conversion in power electronics grows as well. This task is currently met using silicon devices. However, silicon is rapidly reaching its operational limit^2^. Gallium nitride (GaN) has become the topic of much research in building devices which can replace Si power devices. Gallium nitride is preferred due to its high bandgap energy, a high critical breakdown electric field, and a high thermal conductivity^3–5^. Research on many GaN devices such as vertical GaN p-n diodes, p-n diodes with avalanche capability, and vertical transistors has been done^6–8^ and shows the need for consistently doped GaN layers to form these devices. All three of these devices rely on a thick (upwards of 180 µm) layers of N^+^ doped GaN. This has been an issue with several other doping methods, especially while maintaining a doping consistent throughout the GaN layer. This research shows the benefits thermal neutron transmutation doping can bring to the doping of GaN.

Previous work in GaN doping comes in four forms, during growth, ion implantation, diffusion, and neutron transmutation doping. Doping during growth has been extensively studied with a wide variety of elements to create both n-type and p-type GaN wafers. GaN was doped with both Si and Ge using GeH_4_ and SiH_4_^9^. The growth was made by first creating a buffer layer of GaN and then growing the doped layer using two-flow metalorganic chemical vapor deposition (MOCVD). While the surface of the Si doped layers was exceptional the surface of the Ge doped layers contained pits at higher carrier concentrations. Similar results were found by using a hydride vapor phase epitaxy^10^. Initial experiments used hydrogen as the carrier gas, which led to a pitted surface on the GaN. Using nitrogen as the carrier gas instead of hydrogen removed the pits. However, it led to a hillock surface in the grown crystal. Later experiments used a buffer layer of undoped GaN before the doping process^11^. Silicon doping of the GaN material significantly affected the surface and degraded the quality. Selenium doping was attempted using low pressure metalorganic chemical vapor deposition (LP-MOCVD)^12^. This method worked but resulted in surface degradation at higher concentrations.

Ion implantation with GaN typically involves bombarding the surface of the GaN with the ion needed and then annealing the GaN to redistribute the ions throughout the crystal structure. A wide variety of elements have been tested using ion implantation^13^. While many elements showed redistribution when annealed at 600 °C, Ge did not show redistribution even at 900 °C. Experiments studying ion implantation of GaN with Si, Mg, and Ca found that after implantation the sheet resistance of the GaN was on the order of 10^5^ Ω/sq.^14^. The GaN was then annealed to electrically activate the dopants. The sheet resistance only dropped for materials that were doped with Si at 1050 °C, in the range of 10^3^ Ω/sq. For the Mg and Ca doped material, the sheet resistance varies widely between 10^4^ Ω/sq. and 10^7^ Ω/sq. at various annealing temperatures.

Diffusion of dopants in GaN has been attempted using a few different approaches. In this method of doping, the dopant element diffuses through the semiconductor at elevated temperatures. The dopant layer may be introduced by sputter coating the dopant onto the semiconductor surface and then heating the semiconductor. When this method was attempted using a sputtered layer of Mg on top of GaN, the layer was then capped with either a metal or silicon oxide and then heated to either 850 °C or 900 °C for 6 hours^15^. The results of these experiments showed that the diffusion doping was possible, but it relied heavily on the capping layer. In addition to the variation with the capping layer the experiment showed some variability in GaN wafer with the same testing conditions. This implies that the doping is heavily dependent on the structure of the undoped GaN. Attempts to improve this process included first growing undoped GaN around three Mg “spikes” and then annealing the structures at 1060 °C for 1.25 h^16^. The results of this experiment showed little diffusion of the Mg through the GaN. Another attempt to improve the diffusing process of Mg into GaN was to grow a Mg doped GaN layer on an undoped GaN at temperature between 925 and 1050 °C^17^. Although the results are encouraging, the doping profile is typical of the diffusion doping process and not uniform across the depth of the wafer. Differently from various approaches described above, GaN can be doped using the neutron transmutation doping (NTD) process when it is placed in a high flux neutron field. The necessary irradiation conditions require a nuclear reactor and irradiations are typically done at research reactor user facilities.

Neutron transmutation doping is a method of semiconductor doping which was first demonstrated in 1961^18^. There, the process was used to transmute silicon in phosphorus. This resulted in n-type doped silicon with an extremely uniform doping profile. This is due to the extremely even flux profile of a nuclear reactor. The process required the annealing of the silicon at 600 °C but resulted in a method of bulk doping silicon which is widely used in nuclear reactor facilities today. Several groups have also reported on NTD of GaN. The first results of NTD in GaN came from a study of fast neutron damage in GaN and in n-AlGaN∕GaN heterostructures^19,20^. The GaN material was irradiated under a Cd cover to filter thermal neutrons and pass epithermal and fast neutrons. The authors reported that neutron damage created a semi-insulating GaN material. In a subsequent experiment the GaN material was irradiated without a Cd filter in a research reactor irradiation position reported to have a 1:1 thermal:fast neutron ratio^21^. This experiment also produced an insulating GaN substrate. The resistivity of the GaN dropped from the 10^6^ Ωcm range to the 1 Ωcm range after annealing at 1000 °C. Electron hole traps were present in the material post annealing. Other experiments irradiated GaN in an irradiation position with a thermal:fast neutron flux ratio of nearly 2:1^22^. Significant displacement of the GaN in the <0001> row was observed. The samples were annealed and a photoluminescence measurement showed the presence of lattice damage. Subsequent experiments conducted a test using a 1:1 thermal to fast neutron dose and found this created many electron traps that were not mitigated by annealing at 1000 °C^23^. The trap density was reported to increase with increased Ge levels implying that the creation of the traps is caused by radiation damage. Experiments on the NTD of GaAs showed an increased thermal:fast ratio reduced radiation damage^24^. This new experiment relies on using a primarily thermal neutron flux in order to avoid the extreme damage encountered in previous experiments. Thermal neutron flux is lower energy than fast neutron flux and is less likely to damage the crystal structure of the GaN.

## Theory

The number of captures per unit volume, N, is described by:

$${\text{N}} = {\text{N}}_{{\text{T}}} \sigma_{{\text{c}}} \Phi$$

where N_T_ is the number of target nuclei per unit volume, σ_c_ is the capture cross section with units of cm^2^ and Φ is the neutron fluence, n/cm^2^. The cross section represents the probability of interaction between the neutron and the nucleus. Neutron capture reactions occur at higher rates with low energy neutrons because the lower velocity allows greater interaction with the target nuclei. The capture cross section is inversely proportional to the neutron velocity at low energies. Reactor neutrons are parameterized into three groups based on energy; fast neutrons have greater than 100 keV, epithermal neutrons have between 0.5 eV and 100 keV, and thermal neutrons have less than 0.5 eV with a most probable energy near 0.025 eV. Especially, NTD of GaN occurs when ^69^Ga, ^71^Ga, and ^14^N undergo neutron capture reactions with thermal and epithermal neutrons to produce unstable ^70^Ga, ^72^Ga, and ^14^C, respectively. The ^70^Ga and ^72^Ga have half-lives of 21.1 min and 14.1 h and beta decay to ^70^Ge and ^72^Ge, respectively. The ^14^N neutron capture reaction is exothermic and produces ^1^H^+^ in the lattice leading to proton knock-on damage. Fast neutrons also cause knock-on damage through elastic scatter.

Previous research focused on irradiation which had equal or nearly equal thermal to fast neutron fluxes. In this work, NTD of GaN was conducted in the hanging wedge regions of the University of Missouri Research Reactor (MURR) with a thermal to fast neutron flux ratio of 25:1. This irradiation position was expected to reduce radiation damage caused by fast neutron knock-on damage compared to previous studies.

## Experiment

### Irradiations

The GaN wafers and neutron flux monitors were placed inside of an Al capsule with two flux wires. The capsule was sealed inside of a welded Al can and leak tested. The thermal neutron flux was monitored using NIST SRM 953 cobalt in aluminum neutron density wire and the fast neutron flux was monitored using nickel wire. During irradiation, the sample can was rotated to minimize variation in the axial neutron flux profile. The wafer thickness was 350 µm and changes in the axial flux profile were considered insignificant. Following irradiation, the GaN wafer was decayed for ~ 10 days and the activity of ^72^Ga (T_1/2_ = 14.1 h), ^60^Co (T_1/2_ = 5.27 y), and ^58^Co (t_1/2_ = 70.86 d) were measured by gamma ray spectroscopy using a high purity germanium detector (HPGe) running Canberra Genie 2000 software. The dead-time for the measurements was less than 10% and was corrected using a live-time algorithm. The HPGe detector was calibrated using a NIST traceable multi-gamma source purchased from Eckert and Zeigler. The atom density of ^72^Ge was determined from the measured ^72^Ga activity.

The ^70^Ga activity cannot be measured directly using gamma ray spectroscopy because of its short half-life. However, we can have a theoretical calculation using the measured ^72^Ga activity. The production of ^70^Ge is calculated based on the neutron flux and the nuclear cross sections of the neutron capture reaction of ^69^Ga. This gives a theoretical value for the production of ^70^Ga. The same theoretical calculation is also used for the ^72^Ga production. The ^70^Ge concentration can be estimated from the ^72^Ge assuming that the theoretical ratio of ^70^Ge and ^72^Ge concentration is the same with the experiment one. The ^70^Ge concentration is later confirmed with SIMS measurement.

Four irradiations were conducted for this research with varying fluxes and irradiation times. These parameters are shown in Table 1. Experiment #1 was the initial test with 1 cm × 1 cm wafers. Experiment #2 increased the irradiation time and changed the flux to higher thermal neutrons percentage using 1 cm × 1 cm wafers. Experiment #3 increased the irradiation time to 600 h to achieve 1.06 × 10^18^ Ge atoms/cm^3^, also using 1 cm × 1 cm wafers. Experiment #4 used similar irradiation parameters to Experiment #2 but used sealed containers to protect the surface of the samples from the water in the reactor and resulted in doped 1 cm × 1 cm wafers and one 2″ wafer.

**Table 1:** Experimental parameters.

Experiment number	Thermal flux (Neutrons/cm^2^ s)	Resonance flux (Neutrons/cm^2^ s)	Fast flux (Neutrons/cm^2^ s)	Irradiation time (h)	Ge atoms/cm^3^
#1	5 × 10^12^	1.83 × 10^12^	1.67 × 10^11^	4.6	1 × 10^16^
#2	3.76 × 10^12^	3.76 × 10^10^	1.52 × 10^11^	60	1.2 × 10^17^
#3	3.76 × 10^12^	3.76 × 10^10^	1.52 × 10^11^	600	1.06 × 10^18^
#4	3.01 × 10^12^	3.01 × 10^10^	1.22 × 10^11^	60	8 × 10^16^

### Annealing process

**Figure 1:**
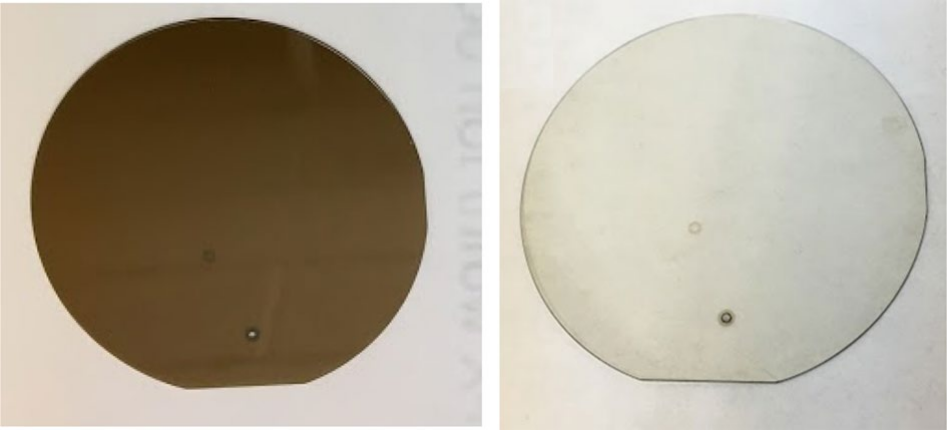
A 2″ GaN wafer after irradiated for 60 h (left) and after the annealing process (right).

After irradiation the wafers suffer from discoloration, as shown in Fig. 1 (left). Before irradiation the GaN wafers are clear, and the annealing process helps restore this clarity, as shown in Fig. 1 (right).

The annealing process uses a stainless-steel pressure chamber which is filled with ultra-pure nitrogen gas. The chamber is pressurized to 331 kPa. The chamber is then heated to 950 °C. At this point the pressure of the nitrogen gas is 1.38 × 10^3^ kPa. This pressurized environment is necessary to avoid surface degradation of the GaN as at this temperature the nitrogen in the GaN begins to break off from the crystal structure and the GaN decomposes. The pressurized nitrogen environment prevents this from happening.

## Results and discussion

### Concentration measurements

**Figure 2:**
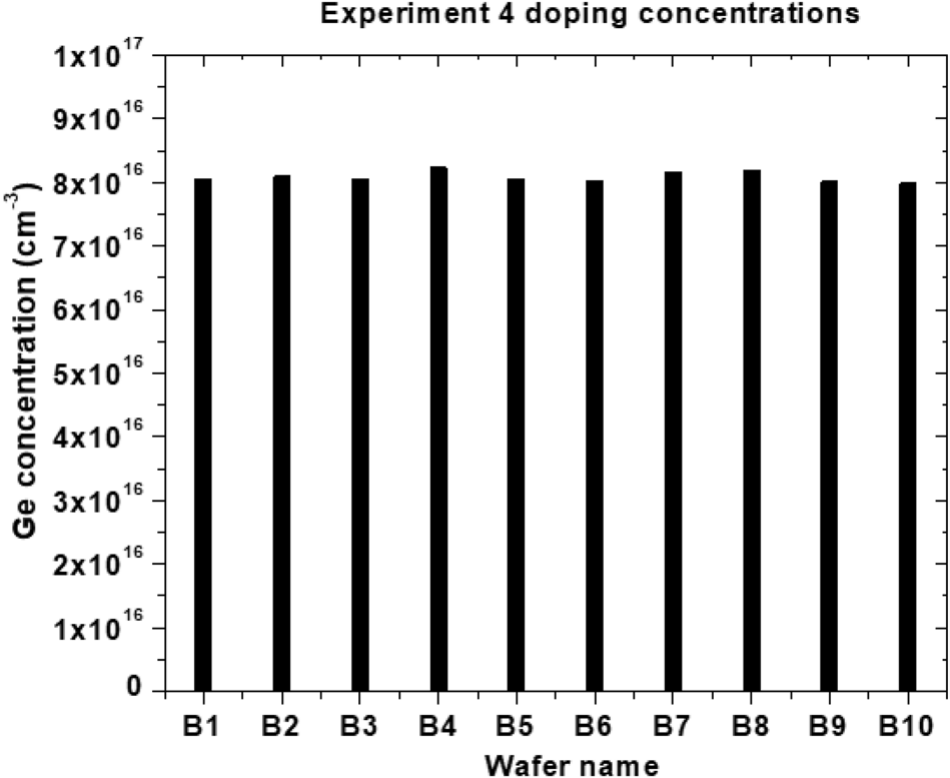
Ge concentrations of the 10 of 1 cm × 1 cm precut samples from B wafer after Experiment #4.

As detailed in the section III.A above, Fig. 2 shows the gamma-ray spectroscopy measurement data for germanium concentrations of precut samples from the wafer B, which was irradiated in Experiment #4.

The germanium concentration of A and C samples are very similar to B samples. Wafers A1-A10 and B1-B10 are the 1 × 1 cm pieces which were arranged to emulate a 2″ wafer while wafer C was a 2″ wafer. The highest concentration comes from the 2″ wafer with 8.47 × 10^16^ Ge atoms/cm^3^ and the smallest comes from B10 with a 7.98 × 10^16^ Ge atoms/cm^3^. This shows a variation of 5.7%. This difference in concentrations may come from a difference in geometry influencing the gamma count. Wafer C is significantly larger than the others and Wafer B10 was broken during the irradiation process.

In order to confirm the gamma-ray spectroscopy measurements, two other type of measurements were also conducted, ICP-MS and SIMS. Since the laser ablation feature of the ICP-MS was not available, another method to dissolve the GaN had to be used. A process known as borate fusion was employed to break down the GaN into solid borate flux, which could then be easily dissolved in a mineral acid. The dissolved samples are compared against pure gallium standards and pure germanium samples. A concentration of 7 × 10^16^ ± 2 × 10^16 72^Ge atoms/cm^3^ was measured in one of the samples from Experiment #2. The reason for the large error in this measurement is because of how similar the atoms being measuring against are. ^72^Ge and ^71^Ga are only one proton different. In the plasma it is possible for the ^71^Ga to combine with a hydrogen atom to for a gallium hydride. Since ICP-MS measures based on mass size, this means that a ^72^Ge and a ^71^Ga with a hydrogen atom bonded will be measured as the same atom. This problem is even worse for the ^70^Ge measurements. There is an additional polyatomic, ^40^Ar_16_O_14_N + , which forms in the plasma and has a mass of 70. Since this test is destructive (the GaN wafer is completely dissolved) and cannot measure with a reasonable amount of certainty, gamma-ray spectroscopy was chosen as the preferred measurement method.

**Figure 3:**
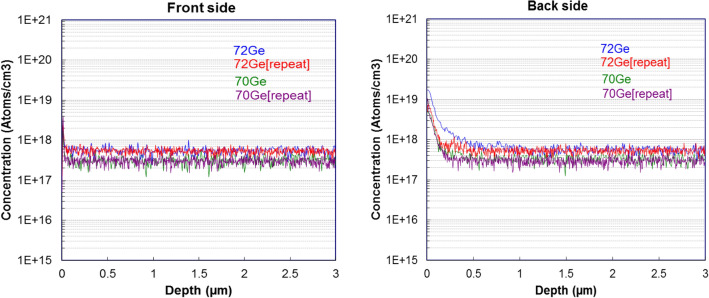
SIMS data showing consistent doping concentration of both ^70^Ge and ^72^Ge in a 10^18^ doped GaN wafer.

One information gamma-ray spectroscopy cannot provide, however, is the doping concentration profile. To measure this 10^17^ Ge atoms/cm^3^ concentration doped GaN wafers and 10^18^ Ge atoms/cm^3^ concentration doped GaN wafers were sent to a partner, EAG Laboratories, for SIMS analysis. Figure 3 shows the results of two measurements on the 10^18^ Ge atoms/cm^3^ concentration doped wafer. This SIMS analysis not only helps confirm the estimations used in the gamma-ray spectroscopy, but it also shows how consistent the germanium doping concentration is throughout the wafer. This is to be expected as the neutron flux drop-off through the thickness of the wafer (350 µm) is extremely small. Calculation using the thermal cross sections for ^71^Ga and ^69^Ga (4.61 barns and 1.68 barns respectively) shows that the flux reduction across the 350 nm thickness of GaN wafer is 7.7 × 10^–5^%.

### UV–Vis measurement

UV–Vis spectroscopy measurements were conducted on the GaN pieces to better quantify the annealing results. This test involved shining light through the samples and recording the transmittance of wavelengths from 200 to 700 nm. Before irradiation GaN begins as a mostly clear crystal. After irradiation there is damage to the crystal which causes a brown coloration, as shown in Fig. 1. Other from transmuting Ga to Ge and N to C, the radiation process also damages or produces defects in the GaN lattices. The primary defects generated from the irradiation process (neutron or gamma) are Frenkel pairs, a pair of a vacancy and an interstitial defect, such as nitrogen vacancies, nitrogen interstitials, Ga vacancies, and Ga interstitials. The UV–Vis spectra were used to quantify these damages. The significant drop of the transmittance across the visible spectrum shows the presents of many of these defects at different energy levels. After the annealing process, most of these defects were removed and the samples become transparent again.

**Figure 4:**
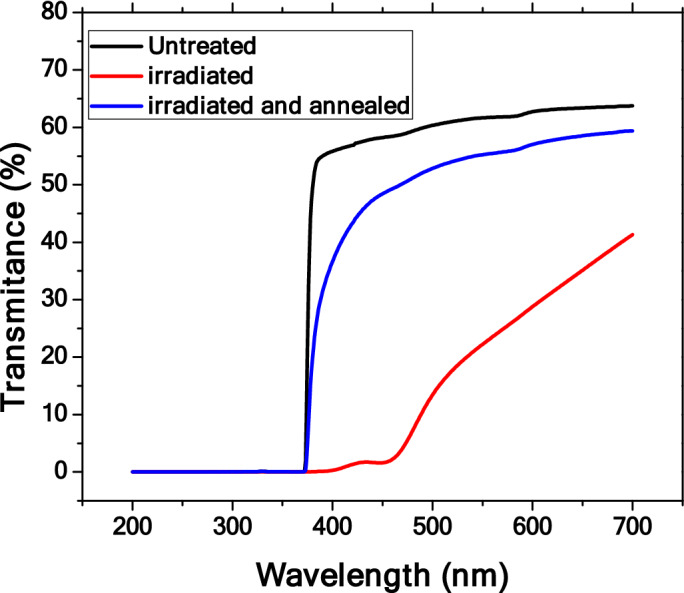
The UV–Vis spectra of GaN-B1 before irradiation, after irradiation, and after annealing.

The UV–Vis spectrum of a GaN wafer before being treated is the black curve in Fig. 4. The UV–Vis spectrum of the sample after irradiation process is represented by the red curve and after irradiation and annealing is represented by the blue curve. This measurement was conducted on each of the GaN pieces irradiated and annealed, and Fig. 4 shows the results for one of these pieces, GaN-B1. The red curve (irradiated) shows the issue of the brown discoloration of the wafers. In the 375 nm to 475 nm range the transmittance drops to nearly 0% from 60% of the untreated sample. Even at the 700 nm wavelength the difference in transmittance goes from 65 to 40%. After annealing there is a significant improvement. The 375–475 nm range is no longer at 0% transmittance but is instead much closer to the untreated GaN.

### C-V measurements

**Figure 5:**
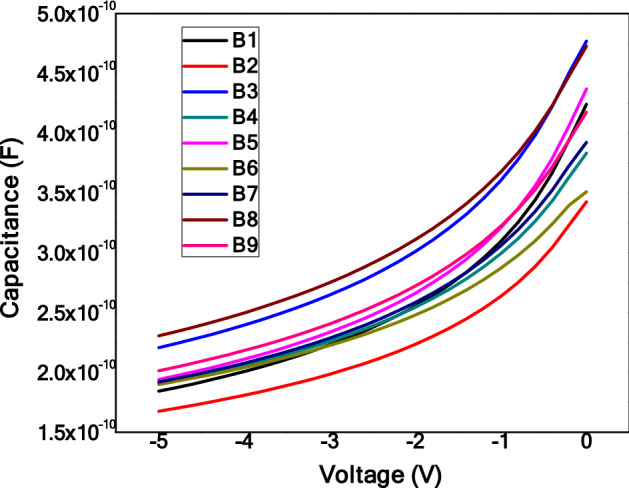
The C-V measurements of GaN 1–10 using mercury probe.

**Figure 6:**
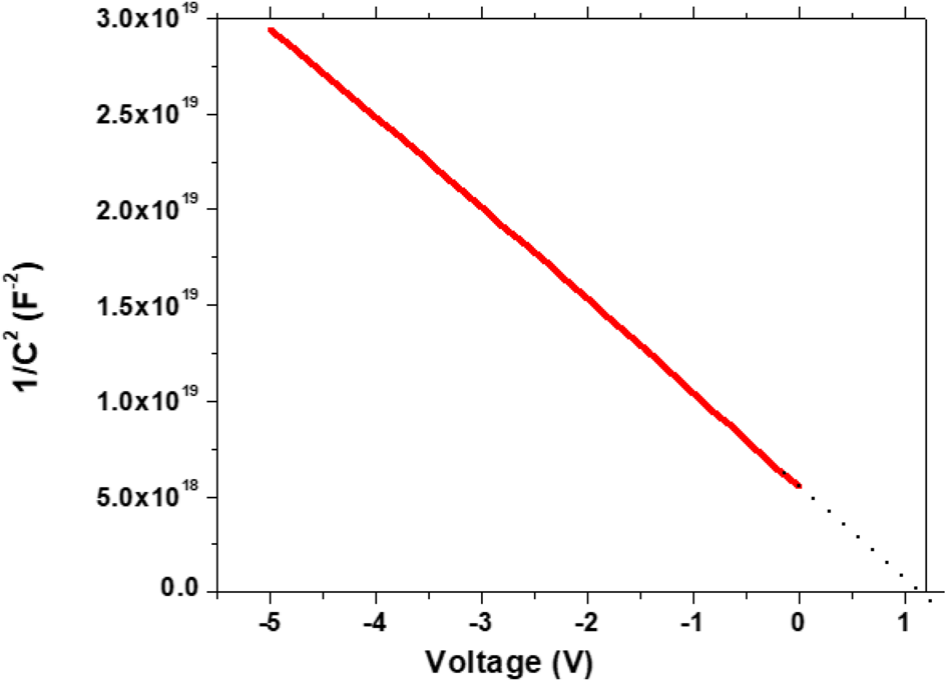
The 1/C^2^ vs V plot of the GaN-1 wafer.

Another method employed to characterize the GaN wafers was C-V measurements. To conduct these measurements, a Hg probe was used. The mercury probe uses a vacuum system to create a ring and a dot contact on the surface of the GaN wafer. The back contact of the probe is made of stainless steel. The measurements were done with a voltage sweep from − 5 to 0 V with steps of 0.1 V. Bellow in Fig. 5 are the C-V measurement curves for GaN B1- 9. The graph of 1/C^2^ for the C-V measurements in Fig. 6 is important as it allows for the calculation of the carrier concentration of the wafer.

### Consistent carrier concentration

To calculate the carrier concentration from the C-V measurement results, the following equation is used.

$$\rho = \frac{2}{{A^{2} q\varepsilon_{0} \varepsilon m}}$$

**Figure 7:**
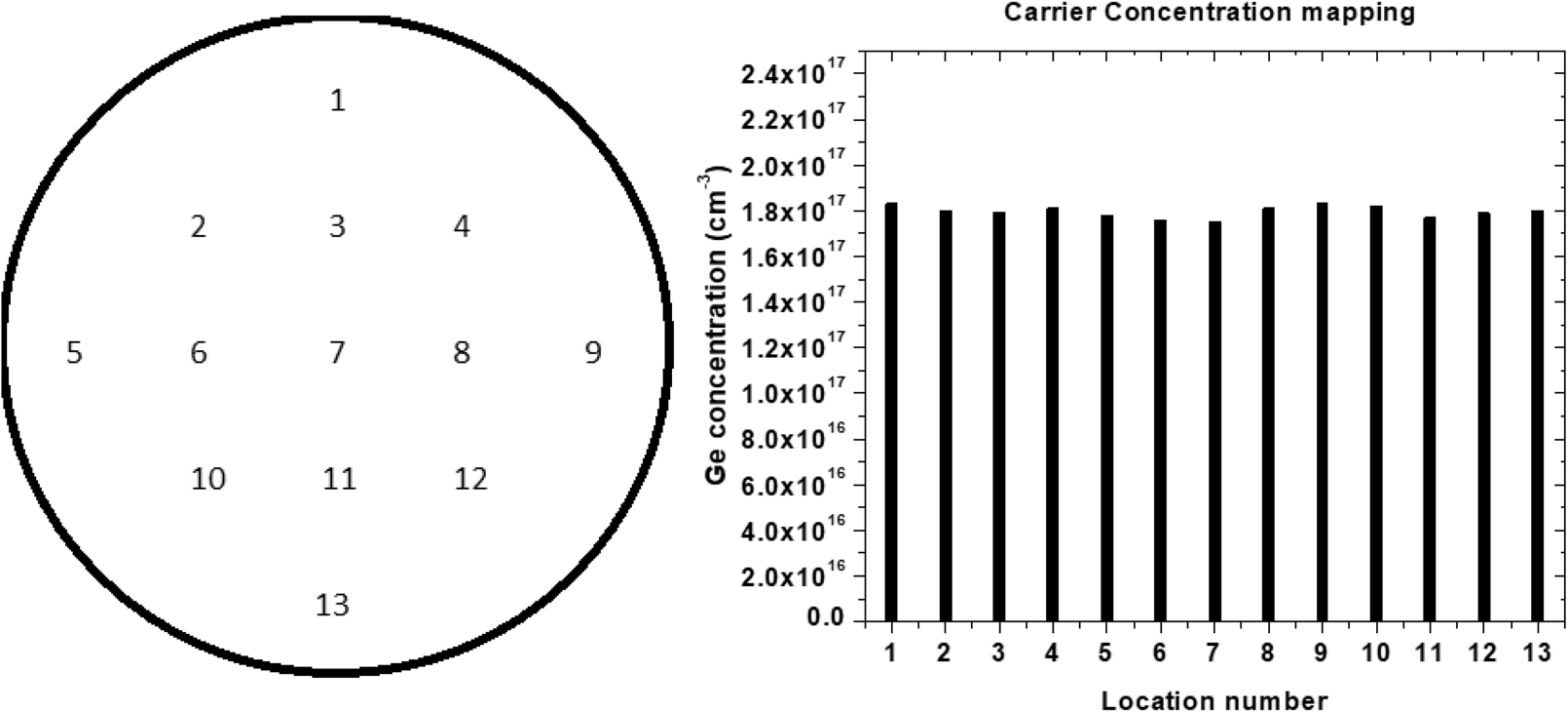
The carrier concentration of each location on wafer C.

In this equation ρ is the carrier concentration, A is the area of the probe contact (0.00398 × 10^–4^ m^2^), q is the charge of an electron (1.6 × 10^–19^ C), ε_0_ is the permittivity of space (8.854 × 10^–12^ F/m), ε is the permittivity of GaN (8.9) and m is the slope of the 1/C^2^ curve. The main goal was to prove a consistent carrier concentration across a 2″ wafer of GaN. To do so C-V measurements were conducted across 13 points on wafer C. The location of these measurements and the data from them are shown in Fig. 7 below.

When comparing the four methods of measuring doping concentration, ICP-MS, gamma-ray spectroscopy, SIMS, and C-V there are two clear favorites. ICP-MS is by far the least accurate method as the dopant atoms are merely one atomic mass unit larger than the atom they are replacing which makes ICP-MS unreliable. SIMS is effective for finding the depth of the doping concentration; however, it has its limitations. The results for the 10^17^ Ge atoms/cm^3^ are not shown in this paper as the concentrations at that doping range are near the minimum possible to measure using SIMS. While this is not a problem for the 10^18^ Ge atoms/cm^3^ samples, the 10^17^ Ge atoms/cm^3^ samples are the ones being heavily produced in this study.

Gamma-ray spectroscopy appears to be the best methods available to us to measure the germanium concentration. Gamm-ray spectroscopy relies on the theory behind the neutron transmutation to measure the ^70^Ge concentration. This theory has proven to be true after testing with both SIMS and C-V. For C-V measurements there is no way to distinguish between the ^70^Ge and ^72^Ge concentrations, but that isn’t necessarily needed when it comes to measuring doping concentration. Either way, the doping concentrations measured using the two methods seem to be close. The gamma-ray spectroscopy measurement for the 2″ wafer was measured to be 8.47 × 10^16^ Ge atoms/cm^3^. The C-V measurement had the carrier concentration as 1.806 × 10^17^ cm^−3^. The carrier concentration of an untreated GaN wafer is 1 × 10^17^ cm^−3^. This would mean that 8.06 × 10^16^ Ge atoms/cm^3^ were added which is very close to the Ge concentration results from gamma-ray spectroscopy measurement.

## Summary

The irradiation of GaN using mostly thermal neutron results in high quality, consistent and uniform doping throughout the GaN wafer. It has been shown that germanium doping of GaN using primarily thermal neutrons can effectively dope GaN in concentrations from 10^16^ Ge atoms/cm^3^ to 10^18^ Ge atoms/cm^3^. Before the annealing process, the resistivity of these GaN samples was several orders of magnitude below the results of previous research which used equal doses of fast and thermal neutrons. After annealing the resistivity is even further reduced. During the irradiation, the GaN wafer loses a majority of its transparency. But after annealing, this transparency comes almost entirely back. The neutron doping process creates extremely consistent doping across large areas and throughout the depth of the wafer making it a prime candidate for creating large high-quality Ge-doped GaN substrates.
